# Transmission and dose–response experiments for social animals: a reappraisal of the colonization biology of *Campylobacter jejuni* in chickens

**DOI:** 10.1098/rsif.2011.0125

**Published:** 2011-05-18

**Authors:** Andrew J. K. Conlan, John E. Line, Kelli Hiett, Chris Coward, Pauline M. Van Diemen, Mark P. Stevens, Michael A. Jones, Julia R. Gog, Duncan J. Maskell

**Affiliations:** 1 Cambridge Infectious Diseases Consortium, Department of Veterinary Medicine, University of Cambridge, Cambridge, UK; 2 DAMTP, University of Cambridge, Cambridge, UK; 3 Department of Veterinary Medicine, University of Cambridge, Cambridge, UK; 4 USDA, Agricultural Research Service, Poultry Microbiological Safety Research Unit, Athens, GA, USA; 5 Institute for Animal Health, Compton, UK; 6 School of Veterinary Medicine and Science, University of Nottingham, Nottingham, UK

**Keywords:** dose response, transmission, *Campylobacter jejuni*

## Abstract

Dose–response experiments characterize the relationship between infectious agents and their hosts. These experiments are routinely used to estimate the minimum effective infectious dose for an infectious agent, which is most commonly characterized by the dose at which 50 per cent of challenged hosts become infected—the ID_50_. In turn, the ID_50_ is often used to compare between different agents and quantify the effect of treatment regimes. The statistical analysis of dose–response data typically makes the assumption that hosts within a given dose group are independent. For social animals, in particular avian species, hosts are routinely housed together in groups during experimental studies. For experiments with non-infectious agents, this poses no practical or theoretical problems. However, transmission of infectious agents between co-housed animals will modify the observed dose–response relationship with implications for the estimation of the ID_50_ and the comparison between different agents and treatments. We derive a simple correction to the likelihood for standard dose–response models that allows us to estimate dose–response and transmission parameters simultaneously. We use this model to show that: transmission between co-housed animals reduces the apparent value of the ID_50_ and increases the variability between replicates leading to a distinctive all-or-nothing response; in terms of the total number of animals used, individual housing is always the most efficient experimental design for ascertaining dose–response relationships; estimates of transmission from previously published experimental data for *Campylobacter* spp. in chickens suggest that considerable transmission occurred, greatly increasing the uncertainty in the estimates of dose–response parameters reported in the literature. Furthermore, we demonstrate that accounting for transmission in the analysis of dose–response data for *Campylobacter* spp. challenges our current understanding of the differing response of chickens with respect to host-age and *in vivo* passage of bacteria. Our findings suggest that the age-dependence of transmissibility between hosts—rather than their susceptibility to colonization—is the mechanism behind the ‘lag-phase’ reported in commercial flocks, which are typically found to be *Campylobacter* free for the first 14–21 days of life.

## 1. Introduction

*Dose–response* studies are an essential method for the risk assessment of infectious agents [1], characterizing the relative infectiousness of different pathogens [2]. The proportion of hosts that *responds* to inoculation with an infectious agent typically increases with the applied *dose*. The standard interpretation of such *dose–response* data stems from the analysis of simple mathematical models, which provide a probabilistic—and mechanistic—link between the nature of the infectious agent and its method of reproduction within the host and the shape of the observed dose–response curve. Theoretical dose–response models are based on the assumption that the individuals within a particular challenge group (at a given dose) are independent. For social animals such as poultry, health and welfare regulations recommend that animals are housed in groups for experimental studies wherever possible [3]. Group housing is considerably more practical and cheaper than individual housing, however it introduces the possibility that transmission occurs between co-housed hosts. If transmission is allowed to occur during a challenge experiment, the final infection status of an individual host is not only dependent on the applied dose but also on the infection status of the rest of the group.

Group challenge experiments have been used routinely in studies of the dose–response relationship of *Campylobacter jejuni* in chickens [4–11] and other infectious agents in avian species [12,13]. Broiler chickens routinely engage in coprophagic activity (oral ingestion of faecal matter), which is likely to enhance the transmission of enteric pathogens when they are housed together. Transmission is therefore likely to play a particularly important role in the interpretation of dose–response data for these hosts [14]. Infection of chickens with *C. jejuni* is thought of as being commensal, leading to colonization which is typically persistent over the timescales of challenge experiments [4].

Taking *C. jejuni* as our study system, we explore the impact that transmission between co-housed hosts has on the observed dose–response relationship for an epidemic process where no recovery from infection occurs. We construct a joint likelihood for the response of a group of challenged hosts by combining standard dose–response models with a *susceptible–infective* (SI) transmission model [15]. Although the simultaneous estimation of dose–response and transmission processes is possible, we show that individual housing is the optimal design for the estimation of dose–response parameters. We apply our model to re-analyse published dose–response data for *C. jejuni* in chickens and demonstrate that the proper accounting for transmission radically alters the interpretation of these data.

## 2. Independent action dose–response models

In this section, we shall briefly review the theory of independent action dose–response models with a particular emphasis on the theoretical interpretation of the shape of empirically derived *dose–response curves*. Exposure of a host organism to an infectious particle does not always result in infection, with the probability of infection increasing with the challenge dose. Mechanistic dose–response models describing this relationship are based on two mutually exclusive hypotheses. *Co-operative action models* postulate the existence of a *minimum infective dose* with infection requiring the cooperation of a population of infectious particles [16]. Although there exist some notable exceptions, such as multi-component plant viruses [17–19], the alternative *independent-action* hypothesis [20] is more generally supported [21–24], although direct experimental validation has only been attempted for a handful of host–pathogen systems [25,26].

Probabilistic independent-action models are particularly appealing theoretically as they provide a biological interpretation for the shape of empirically derived dose–response curves [27], are simple enough to be fitted directly to experimental data using likelihood-based methods [21,28–30] and allow for the extrapolation of infection risk to low doses [31,32]. Independent-action models follow on naturally from two central assumptions:

— infection can progress from a single infectious particle reaching a favourable site within the host; and
— the probability that a given infectious particle is capable of initiating infection is independent of the dose.

These assumptions facilitate the derivation of simple mathematical relationships between the applied dose (*D*) and the probability of infection *P*_inf_(*D*) in terms of the ‘single-hit’ probability (*r*)—defined as the (non-zero) probability of a single infectious particle initiating infection within a given host [31,32]. The simplest ‘single-hit’ model is formed by assuming that *r* is constant between different infectious particles and hosts. We rarely know the exact number of infectious particles within an experimental inocula. At best, we can characterize the expected number of particles, given the average concentration within our inoculum [27]. Provided that each particle acts independently and 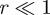, the probability of a host escaping infection given an innoculum with an expected number of ingested organisms *D* is given by the first term of a Poisson series 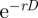. Thus, the probability of infection *P*_inf_ (*D*; *r*) is:2.1
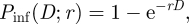
which has naturally come to be known as the exponential dose–response model [31].

The most common goal of dose–response studies is the identification of the minimum effective infectious dose that will consistently lead to colonization on challenge. This can be characterized in terms of the ID_50_ —defined as the dose at half-height of the dose–response curve, where the chances of infection are 50 per cent (*P*_inf_ (*D*) = 0.5). The shape of dose–response curves is most commonly characterized in terms of the slope at half-height. As empirical dose–response relationships typically range over several orders of magnitude, it is customary to plot dose-curves as a function of the log dose (log_10_(*D*)). We, therefore, calculate the slope at half-height against the log dose: d/(dlog_10_(*D*))(*P*_inf_)|*D* = ID_50_. Equation (2.1) describes a family of sigmoidal curves with ID_50_ = ln(2)/*r* and a constant slope at half-height of (1/2)ln(10)ln(2) ≈ 0.798. Slopes at half-height steeper than (1/2)ln(10)ln(2) therefore violate the hypothesis of independent action and imply cooperative action between the infectious particles [17].

A variety of stochastic factors relating to the survival of the infectious particle and the action of host defences may prevent a particle from successfully invading its host. If *r* varies between individual infectious particles but not between different hosts, then the shape of the dose–response curve is unchanged (with an effective value of *r* equal to the mean of the population of particles). However, any variation in *r* between hosts will modify the shape of the dose–response relationship leading to slopes at half-height shallower than (1/2) ln(10)ln(2) on the log_10_-scale [33]. We can generalize equation (2.1) by describing the variation in *r* between hosts by a distribution *f*(*r*), then:2.2
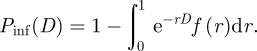


In principle, the form of *f*(*r*) can be modelled in terms of the interaction between the infectivity of infectious particles and the susceptibility of the host population [27]. In practice, we must assume a functional form in order to fit dose–response models to data. The hypergeometric dose–response model arises from assuming that *r* takes the form of a Beta distribution and takes the form:2.3
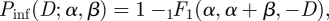
where _1_*F*_1_ is the Kummer confluent hypergeometric equation of the first kind, which must be evaluated numerically. The hypergeometric model takes two shape parameters (*α*, *β*), describing a family of curves with a slope at half height, which ranges up to the single-hit limit of (1/2)ln(10)ln(2) and is determined by the variability of the single-hit distribution *f*(*r*).

## 3. Methods

### 3.1. Impact of transmission on the dose–response relationship

The basic biology captured by dose–response experiments can be summarized in terms of two statistics. The ID_50_ quantifies the scale of the dose–response and the relative *infectivity* of different infectious agents, while the slope at half-height provides a measure of how *susceptibility* varies between hosts. Both of these quantities can be qualitatively assessed, without recourse to explicit mathematical dose–response models, through logistic regression or graphical methods that plot the proportion of responding hosts against applied dose [23]. Transmission between co-housed hosts will lead to systematic biases in both of these quantities. Transmission necessarily leads to the observation of a greater number of colonized animals at all doses, producing an *observed response* (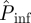), which is both steeper and has a lower ID_50_ than the ‘true’ *individual response*. In this section, we first consider the impact that transmission has on inference based upon naive application of individual dose–response models, before deriving a new mechanistic model for group dose–response data.

**Figure 1.**
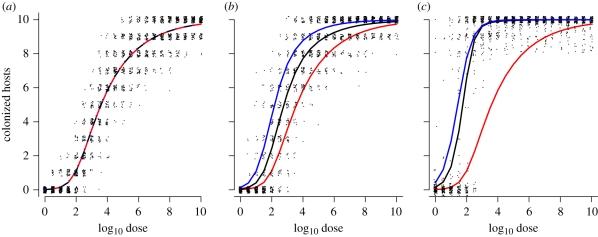
Simulated impact of transmission on dose–response curves. Simulated outcome of 200 replicate dose–response experiments performed in groups of *N* = 10 hosts with increasing levels of within-group transmission *τ* = 0, 1, 5 for panels (*a*–*c*), respectively. The individual dose–response relationship is assumed to be hyper-geometric with *α* = 0.20, *β* = 125, with the expected number of colonized hosts in the absence of transmission (*NP*_inf(*D*)_) given by the solid red lines. The (raw) outcome for each dose group is plotted as a (jittered) dot-density. The observed dose–response curve 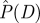 (black lines) and the naive maximum-likelihood estimate neglecting transmission (blue lines) are also shown, which coincide with the individual response curve (red lines) for *τ* = 0. As transmission increases, the observed dose–response develops a characteristic ‘all-or-nothing’ response (*c*) which has been observed experimentally for *C. jejuni* in chickens.

In figure 1, we illustrate the effect of transmission numerically by sampling from a hyper-geometric dose–response model and simulating transmission using a stochastic (SI) epidemic model. The epidemic model is realized as a continuous time Markov process [34] with a single event—transmission—which occurs at a rate given by frequency-dependent transmission: *β*_T_(*N* − *C*(*t*))*C*(*t*)/*N*, where *β*_T_ is the daily transmission rate per infectious animal [35], *C*(*t*) is the number of colonized birds at time *t* and *N* is the group size into which hosts are housed. *t* can be interpreted as measuring the time over which hosts responding to the original challenge dose have the opportunity to transmit the infectious agent to co-housed hosts. Standard dose–response data provide no information on the duration of the latent period between inoculation to infectiousness. We therefore must consider *t* as an unobserved variable within our model with an upper bound imposed by the duration of contact between hosts post challenge. Since *β*_T_ and *t* combine linearly in the solution to the stochastic SI model (equations (3.4) and (3.5)), the impact of transmission can be characterized by a single parameter *τ* = *β*_T_t, which we will refer to as the *total transmission*.

As a binary outcome, we expect to see considerable variation in the observed colonization response for small groups, even in the absence of transmission [22]. Figure 1*a*, with *τ* = 0, demonstrates the extent of this variability for a group size of 10 hosts over a finely grained range of doses. In the absence of transmission, the expected response for dose *D* delivered to a group of size *N* is simply *NP*_inf_(*D*) (black lines, figure 1). Introducing a moderate amount of transmission (*τ* = 1) generates a response that is skewed to lie above the expected individual response (red line, figure 1*b*). As transmission increases, the effect becomes more pronounced leading to a distinctive ‘all-or-nothing’ response (*τ* = 5) (figure 1*c*).

As previously argued, we would expect graphical methods—and regression models—to demonstrate an observed response (black lines, figure 1) which is steeper and with a lower ID_50_ than the individual dose–response (red lines, figure 1). When presented with dose–response data, which have been carried out in identically sized groups, it is not possible to distinguish graphically between the biases in slope and ID_50_ introduced by transmission and the shape of the underlying individual dose–response relationship. Likewise, inference based on individual dose–response models can predict dose–response curves that are inconsistent with the data from grouped experiments. We explore this effect by estimating dose–response parameters by naively applying the (individual) hypergeometric model to the simulated data used in figure 1 and comparing the theoretical (true), observed and maximum-likelihood dose–response curves.

Let *C*(*t*) be the number of colonized hosts in a dose group of size *N* at time *t*. For this simulated dataset, we calculate the observed dose–response curve piece-wise as the ensemble average over realizations at the same dose. Assuming that hosts are housed individually, the probability of colonization at a given dose is constant and given by the individual dose–response relationship *P*_inf_ (*D*; *α*, *β*, ⋯) with parameters (*α*, *β*, ⋯). The probability of observing *l* successful inoculations (*I*) with dose *D* takes the form of a standard binomial likelihood:3.1
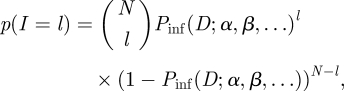
that can be optimized using standard numerical methods to obtain maximum-likelihood estimates for the dose–response parameters (*α*, *β*).

As one would expect, the theoretical, observed and estimated dose-curves coincide for the simulated dataset with no transmission. However, when applied to the datasets with simulated transmission, the ‘naive’ maximum-likelihood estimates (blue lines, figure 1) predict a lower ID_50_ and steeper slope at half-height compared with both the ‘true’ (red lines, figure 1) and the observed dose-responses (black lines, figure 1). For high levels of transmission (figure 1*c*), the slope estimated by an individual dose–response model can be shallower than the observed dose-curve.

Transmission can theoretically lead to ‘observed’ responses with a slope-at-half height steeper than the (1/2)ln(10)ln(2) upper bound of ‘single-hit’ models. However, this model mis-specification is only partly responsible for the failure of the maximum-likelihood estimate to capture the shape of the observed dose-curve. Transmission introduces extra variability to the response between experimental replicates that results in a distribution for (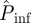) that is over-dispersed (see electronic supplementary material, technical appendix, figure S2) when compared with the binomial model upon which we have based our inference, effectively skewing the fitted mean value at each dose upwards [37]. This bias could be adjusted for using standard methods, such as basing our inference on a Quasi-likelihood or by introducing random or fixed effects that model the degree of over-dispersion with an additional parameter [38]. However, the over-dispersal is the consequence of the significant within-group correlations generated by transmission between co-housed hosts. In the next section, we take a mechanistic approach deriving a correction to the likelihood based on an SI transmission model that links the over-dispersion within the data to the total-transmission (*τ*).

### 3.2. Dose–response experiments in groups

When animals are co-housed each dose group must now be considered as a replicate experiment—rather than each individual animal. The outcome of a challenge experiment in a group of *N* hosts will therefore have *N* + 1 possible outcomes dependent on both the individual dose–response *P*_inf_ and the total transmission *τ*. We can derive the probability of observing a given number of colonized birds in terms of *P*_inf_ and the solution to the stochastic SI transmission model (henceforth HSI: the combined Hypergeometric and SI model). From this distribution, we can calculate the expectation of the combined dose–response and SI model (black lines, figure 1) and the likelihood for a given dataset.

If hosts are housed together within each dose group (of size *N*) then for a given number of colonized birds *C*(*t*), the number of successful inoculations *I* is indistinguishable from the number of birds that acquire the infectious agent through subsequent onward transmission *T*(*t*) with *C*(*t*) = *I* + *T*(*t*). The group dose–response data can therefore be considered to be generated by a hidden Markov model [39], where the observed response *C*(*t*) at the group level is a function of a (hidden) Markov transmission process between individuals. The total probability *p*(*C* = *k*) of observing *m* colonized birds in a group can then be calculated by summing over all of the possible (hidden) values of the unobserved state-variables *I* + *T*(*t*) = *C*(*t*):3.2
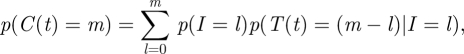
where *p*(*T*(*t*) = (*m* − *l*)| *I* = *l*) is the conditional probability of (*m* − *l*) hosts being colonized via transmission by time *t* given *I* successful inoculations.

Providing recovery does not occur over the course of the experiment, we can model this conditional probability using the stochastic SI model introduced in §3.1. The state-space for the SI model is the number of colonized birds *C*(*t*) = (0,1,2,3, … , *N*) and the probabilities *s*_*i*_ = (*s*_0_, *s*_1_, *s*_2_, *s*_3_, … , *s*_*N*_ ) of occupying state *i* = (0,1,2, … , *N*) satisfy the forward Kolmogorov equations:3.3
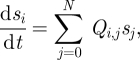
with generator matrix:3.4
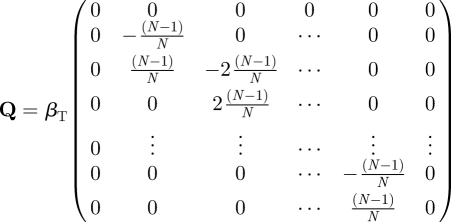
and solution:3.5
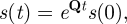
where *s*(0) is the initial state at time *t* = 0. The conditional probability *p*(*T*(*t*) = (*m* − l)|*I* = l) is then simply an element of the matrix 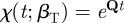:3.6
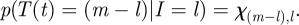


Substituting equations (2.3) and (3.6) into equation (3.2) gives our final expression for the probability of observing *m* colonized hosts in a group dose–response experiment that we will refer to as the solution to the HSI model (*p*_HSI_):3.7
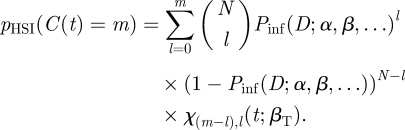


We can express equation (3.7) as a function of *D* and parameters *α*,*β*,*τ* : *p*(*C*(*D*; *α*,*β*,*τ*) = *m*). The expected response E(*C*(*t*)) in a group of N at time *t* will then be:3.8
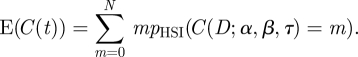


The likelihood for a dataset with a series of doses *D*_*i*_ , group sizes *N*_*i*_ and colonized hosts 𝒞_*i*_ at a series of time-points *t*_*i*_ will be:3.9
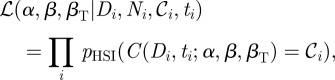
or in terms of the total transmission:3.10
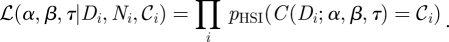


We have now assembled the theoretical framework necessary to estimate dose–response parameters conditional on a background level of within group transmission. In the next section, we apply our combined dose–response and transmission model to infer the level of within-group transmission within three previously published dose–response datasets for *Campylobacter* in chickens and the implications that transmission has for the interpretation of these data. The first dataset [14] we analyse was generated by experiments specifically designed to compare the efficiency of individual and group housing and allows us to support an estimate of transmission using individual dose–response data. In our second analysis, we apply our model to data generated by Ringoir *et al.* [11] designed to compare the dose–response of 2 day and 14 day old chickens for a single isolate of *Campylobacter*. This unique experiment monitored the time series of infection of hosts housed in groups of five over the course of 21 days using cloacal swabbing providing additional information, and challenges, to estimate the level of within-group transmission. Finally, we re-visit the first published study to parametrize mathematical dose–response models for *Campylobacter* in chickens [30]. This study pooled data from a range of published, and previously unpublished, challenge studies carried out in groups of 10 birds. Chen *et al.* compared the dose–response of two groups of *Campylobacter* isolates using a standard dose–response modelling framework. We explore how accounting for transmission modifies their conclusions and the ability to infer differences in the dose–response of these two groups of isolates. We conclude §4 with a final theoretical exploration of the impact that transmission has on the amount of information we can obtain from a dose–response experiment. We quantify how the number of replicate experiments increases with group size and transmission rates, and demonstrate that individual housing is always an optimal experimental design for the estimation of dose–response parameters.

## 4. Results

### 4.1. Comparison of individual and group housing

As a proof of concept for the estimation of transmission from dose–response data, we begin with a re-analysis of a recently published experimental dataset that provides a direct comparison of the suitability of individual and group housing for the characterization of the ID_50_ for *C. jejuni* in chickens [14]. Day-old chicks were inoculated with *C. jejuni* RM1221 and housed either individually—in modified rat cages designed to minimize the potential for transmission—or in groups of 10 in standard isolator units with covered flooring to maximize the potential for transmission. Seven days post-inoculation, the birds were tested for *C. jejuni* colonization by post-mortem caecal sampling.

**Figure 2.**
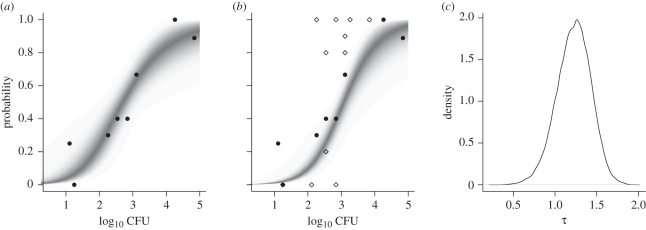
Estimate of transmission for *C. jejuni* in a day-of-hatch chick model. (*a*) Dose–response data for a day-of-hatch chick model carried out in individual housing (filled circles). The posterior predictive distribution (PPD) for the hypergeometric dose–response model is plotted as a density strip where the intensity of shading is proportional to the probability density at that point [36]. (*b*) Dose–response for additional experiments carried out in groups of 10 birds (open diamonds) using the same day-of-hatch chick model was used to parametrize the combined dose response and SI model. The PPD for the individual dose–response relationship based on both the individual and group data is plotted as a black density strip. (*c*) Posterior distribution for the total transmission *τ* estimated from these data. Posterior estimates were calculated from 20 000 samples from the posterior distribution generated by Markov chain Monte Carlo (MCMC) with uniform priors for *α*, *β* and *τ*, with strip-densities estimated using 3000 samples from this posterior distribution.

Graphically, the individual data suggest a markedly shallower response than the group housing data (figure 2). The group data exhibit considerable variation in the number of animals responding at similar doses, demonstrating the ‘all-or-nothing’ response typical of previously published response data from chickens [7] and consistent with the simulated impact of transmission described in §3.1.

In the preliminary analyses of these data, we naively fitted ‘single-hit’ dose–response models to the individual and group responses, details of which can be found in Line *et al.* [14]. Estimates of the ID_50_ based on the hypergeometric model were, as predicted, higher for the individual data when compared with the group response (2.76 log_10_ colony forming units (CFU) compared with 2.35 log_10_ CFU). This difference was exaggerated in the estimates based on the exponential model (3.47 log_10_ CFU compared with 2.52 log_10_ CFU)—a function of the inability of the exponential model to capture the shallow slope exhibited in the individually housed hosts (figure 2*a*). Although this absolute difference is small for a quantity typically quoted only to single log_10_ accuracy, the group estimate was based on killing over twice the number of hosts (160 compared with 76). When the two replicate experimental groups were treated independently the difference between the estimates of the ID_50_ were just as variable as the differences between the different housing groups (2.03 log_10_ CFU compared with 2.74 log_10_ CFU using the exponential model).

We can now re-visit these data [14] using the HSI model (equation (3.10)) to estimate how much transmission between co-housed animals occurred over the course of the experiment. Standard Bayesian Markov chain Monte Carlo (MCMC) methods, implemented in the GNU R package [40], were used to estimate posterior distributions for individual dose–response and transmission parameters (*α*,*β*,*τ*). Uniform priors were assumed for all three parameters to place full weight on the data [41], and standard diagnostic methods were used to assess convergence. In figure 2, we present the marginal posterior distribution for *τ* (figure 2*c*) and compare the posterior predictive distribution (PPD) for the dose–response curve as estimated from the individual data (density strip, figure 2*a*) against the combined individual and group dataset (density strip, figure 2*b*). Pairwise bi-variate posterior distributions (electronic supplementary material, technical appendix) were used to assess the identifiability of parameters, revealing a strong colinearity between estimates for *α* and *β*—a consequence of the uncertainty surrounding the shape (specifically the slope) of the dose–response curve based on these data. By contrast, the posterior samples of *τ* exhibit no systematic relationship with either dose–response parameter.

At face value, the point estimate (median of posterior distribution) for the total transmission *τ* ≈ 1.1 may appear to be surprisingly low given the celerity with which *C. jejuni* is known to spread. Estimates of the frequency-dependent transmission parameter for *C. jejuni* from commercial flocks are of the order of 1–2 infectious contacts per day [42,43] implying an upper bound for a seven day experiment of *τ* = 14. However, we must be cautious in interpreting *τ*. As we discussed earlier in §3.1, the SI model does not account for the latent period between inoculation of a susceptible host and it becoming infectious to co-housed animals, or the possibility that transmission rates vary over the course of the experiment.

While the introduction of a latent class to an epidemic model is straightforward [15], in this context it would necessitate the estimation of an additional parameter from sparse data. Our aim in this study is to account for the impact of transmission in the most parsimonious way. The total transmission *τ* should be considered as a statistical measure of the transmission accrued over a fixed interval of time and is not directly relatable to population-level estimates from commercial flocks. This is still a useful quantity however, as it allows the comparison between different groups accounting for any further difference in the amount of transmission which has occurred.

Dose–response experiments are often used to compare the impact of different treatments [5,7,9–11,30]. Two key studies for *C. jejuni* in chickens have explored the biological effect of host-age [11] and adaptation of *C. jejuni* to laboratory passage [30] via dose–response relationships. These analyses did not account for the possibility that the treatment in question could also affect the transmissibility of *C. jejuni* as well as the individual dose response. We can now re-visit these datasets using our HSI model. We will explore how accounting for transmission modifies the interpretation of these data and in turn our basic understanding of the life history of *C. jejuni* in chickens.

### 4.2. Age and the transmission of *C. jejuni* in chickens

Animal models of *C. jejuni* infection of chickens that are in general usage can be broadly separated into two types, using either day-of-hatch chicks or older birds of 14–16 days. In commercial flocks, birds of less than two weeks of age are rarely naturally colonized by *C. jejuni* [44]. However, once detected *C. jejuni* spreads rapidly [42,43], saturating the flock over the course of 2–3 days. The origin of the so-called *C. jejuni*-free ‘lag-phase’ [45] observed in commercial flocks is still not fully understood, in particular, the relative importance played by the changing susceptibility of the host [46–48] and the opportunities for challenge [49,50].

Ringoir *et al.* performed an elegant series of experiments comparing the dose–response of day-of-hatch and 14 day-old birds [11]. The authors' original qualitative analysis were limited to demonstrating that 2 day-old chicks require a log_10_ lower dose than 14 day-old birds to consistently achieve colonization [11]. Here, we re-analyse their data quantitatively using the HSI model, casting a new perspective on the role that transmission may play in the origin of the ‘lag-phase’ observed in commercial flocks.

What sets Ringoir *et al.*'s study apart from previous studies is that an attempt was made to monitor the infection status of individual birds over the course of the experiment using cloacal swabs. This time-course information potentially allows us to re-interpret the study as a simple transmission experiment and estimate an effective transmission rate *β*_T_ rather than the total transmission (*τ*). However, such data provide additional challenges and must be treated with caution, as recovery from cloacal swabs is less reliable and more prone to environmental contamination than the gold-standard of post-mortem caecal sampling.

A straightforward approach is to treat successive time-points as independent contributions to the HSI likelihood (equation (3.9)). This method has the advantage that all of the observations will contribute equally to the dose–response and transmission components of the model likelihood. Assuming that errors in cloacal sampling are unbiased and independent of the true infection status of a bird, we would expect that the relative comparison between the two groups should be unbiased. However, this assumption of independence ignores the significant correlations that will exist between successive time points and will necessarily lead to an underestimate of the variability of parameter estimates. The most formally appropriate approach to analyse these data, and incorporating this correlation structure, is to construct a likelihood that links successive time points using the solution of the SI transmission model. The likelihood of observing a number of colonized birds (𝒞_*i*_) after inoculation with dose *D* at sampling time (*t*_*i*_) will be conditional on the number of colonized birds at the previous sampling time *t*_*i*−1_:4.1
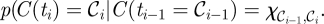


In the absence of information on the duration of latency between inoculation and positivity of cloacal swabs, we must assume that the first sample point (*t*_0_) with positive swabs (𝒞_0_) places an upper bound on the number of colonized birds *I*. This sample point will therefore provide the only likelihood contribution for the combined HSI model, with subsequent observations providing information only on the transmission parameter. The contribution of each dose group of size *N* will then take the form of a product across successive time points:4.2
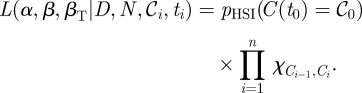


However, the application of equation (4.2) to Ringoir *et al.*'s raw data is not possible without making further assumptions as the time course of swabs (tabulated within the electronic supplementary material, technical appendix) does not form a logically consistent infection history for individual groups. At several time points, the number of positive swabs falls between successive time points. There is no biological evidence to suggest that birds can clear *C. jejuni* over a standard production cycle [4]. Although cloacal swabs are highly correlated with post-mortem status, they are well known to have imperfect sensitivity and specificity. It is impossible to estimate the sensitivity and specificity of cloacal swabs from these data, or distinguish whether a fall in the number of positive swabs is owing to a failure to detect infection at the later time point or the presence of a false-positive result at the earlier time point. However, we can make some progress by reconstructing a logical history of infection based on the extreme assumptions of either perfect specificity or sensitivity of cloacal swabs.

By assuming perfect specificity, we inflate the number of colonized animals at each sampling point potentially leading to an over-estimate of transmission. Likewise, by assuming that cloacal swabbing has perfect sensitivity, we may underestimate the numbers of positive animals and thus reduce our estimate of transmission. These assumptions, therefore, place plausible upper and lower bounds on the transmission rates consistent with these data. By comparison, the assumption of independence is unlikely to introduce a systematic bias into our parameter estimates, but will greatly underestimate the variability in our posterior distributions. To assess the extent to which our assumptions impact on our ability to infer dose–response and transmission parameters, we repeat our inference based on all three of these assumptions and compare the posterior estimates. For brevity, we will refer to these three model fits as *R*1,*R*2, and *R*3 corresponding to the estimates based on the assumptions of independence, perfect specificity and perfect sensitivity, respectively.

**Figure 3.**
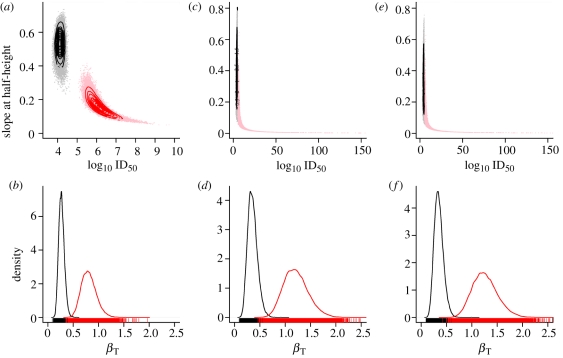
Effect of age on transmission and dose–response of *C. jejuni.* (*a*,*c*,*e*) Comparison of posterior estimates for dose–response and (*b*,*d*,*f*) transmission parameters from data published by Ringoir & Korolik [11] based on treating time points as independent samples (*R*1, (*a*,*b*)) and as a reconstructed logical history of infection assuming perfect specificity (*R*2, (*c*,*d*)) and perfect sensitivity (*R*3, (*e*,*f*)) of cloacal swabs. Posterior distributions are smoothed density estimates (contours and lines) based on 20 000 samples (points) generated by MCMC assuming uniform priors for *α*, *β* and *β*_T_. Dose–response parameters are presented in terms of the bi-variate posterior distribution of the more biologically meaningful parameters: the ID_50_ and slope at half-height on the log scale (*a*,*c*,*e*). (*b*,*d*,*f*) Transmission is presented as the marginal posterior distribution for infectious contacts per day *β*_T_. The qualitative difference between the 2 day-old (black) and 14 day-old chicks (red) is the same—14 day-old birds are more difficult to infect with *C. jejuni* with an order of magnitude greater estimate for the ID_50_, however they appear to transmit *C. jejuni* more efficiently with an estimated transmission rate which is almost twice that for 2 day-old birds. However, treating subsequent time points as independent underestimates, the variability of all parameters, in particular for the ID_50_ of the 14 day group that experienced the greatest rate of transmission. (c) Black line, 2 day; red line, 14 day.

In line with the conclusions of Ringoir *et al.* our analyses based upon the HSI model demonstrate the same qualitative difference in the dose–response relationship between 2 day-old chicks and 14 day-old birds with estimated values for the ID_50_ lower by more than 1 log_10_ for younger birds under all comparisons (figure 3*a*,*c*,*e*). However, the limitations of the approximations we have used to carry out this inference are clear from the differences in magnitude, and variability, of parameter estimates. Treating subsequent time points as independent leads to a lower estimate for the transmission rate for the 14-day birds (figure 3*b*) when compared with estimates based upon a logical infection history assuming either perfect specificity (figure 3*d*) or sensitivity (figure 3*f*) of cloacal swabs. As a consequence, the posterior estimates of the ID_50_ are dramatically more variable based on the logical infection histories, ranging over 150 orders of magnitude (figure 3*c*,*e*) compared with five orders of magnitude based on the independence assumption (figure 3*a*). Despite this variability, we note that the comparison between the two groups of interest is the same under the three different assumptions considered here. Furthermore, this new analysis based on the HSI model suggests that there are biological differences between the two groups that were not immediately apparent from the previous qualitative analysis. The posterior distributions for *β*_T_ are distinct across (*R*1,*R*2,*R*3) with estimated transmission rates for the 14 day-old birds that are approximately three times that of the 2 day-old chicks (point estimates of (0.27, 0.35, 0.34) infectious contacts per day compared with (0.80,1.2,1.2) infectious contacts per day).

Together, these results are at first glance rather surprising suggesting that although 14 day-old birds are more difficult to colonize when challenged with a single low dose of *C. jejuni*, they will subsequently transmit between each other more efficiently once colonization has been established. Given the importance of environmental transmission for *C. jejuni*—mediated through contaminated drinking water and coprophagic behaviour [51,52]—this may simply be a function of the considerably higher doses imparted by natural challenge (with concentrations of *C. jejuni* of up to 10^9^ CFU per gram of caecal matter). Likewise the age-difference may well be a consequence of older birds generating a greater volume of contaminated faecal matter. Another important process that is known to modify the dose–response of *C. jejuni* is that passage through a host results in an adaptive response by the pathogen that increases its ability to colonize and transmit between hosts. This process, which we consider separately in the next section, may also be a function of age, however we cannot make this distinction with the current data.

### 4.3. Laboratory adaptation and the transmission of *C. jejuni* in chickens

*Campylobacter jejuni* demonstrates a remarkable degree of phenotypic and genetic plasticity. The *C. jejuni* genome contains several hyper-variable regions [53], which feature short runs of the same nucleotide (homo-polymeric tracts). These repeats result in a high probability of slip-stranded misalignments occurring during DNA replication resulting in the expression or suppression of these genes [54]. Such changes occur more rapidly than random mutation and if selective pressures are favourable may lead to the rapid ascension of novel phenotypes over the course of colonization of a single host. Dose–response studies have demonstrated that repeated laboratory passage of *C. jejuni in vitro* results in strains, which have a lowered colonization potential *in vivo*, which can be recovered after a single passage through a host [7,10,55].

**Figure 4.**
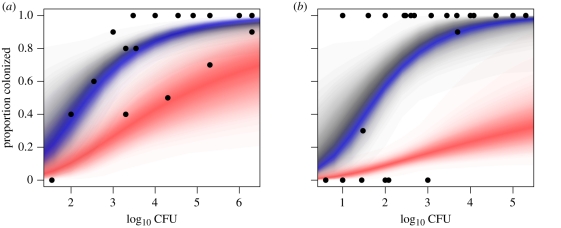
Effect of passage on the dose–response of *C. jejuni.* Comparison of the dose–response of different samples of *C. jejuni* previously analysed by Chen *et al.* [30] and classified as either laboratory adapted ‘lab’ (*a*) or ‘fresh’ (*b*) isolates. Challenge experiments were carried out in groups of 10 birds and colonization status was assessed post-mortem 5 days after challenge. Against the raw data, we compare the PPDs for the observed dose–response (black density strip) and individual dose–response in the absence of transmission (red density strip) to the PPD from naive application of the individual hypergeometric model to these data (blue density strip). Posterior distributions were estimated from 20 000 MCMC samples assuming uniform priors for all parameters, with strip-densities estimated using 3000 samples from these posterior distributions.

Chen *et al.* performed a retrospective analysis to quantify the impact of passage—or rather laboratory adaptation—on the dose–response of *C. jejuni* in chickens [30]. Data were pooled from several published, and previously unpublished, experiments using a range of different strains of *Campylobacter* separated into two broad categories: ‘lab’ isolates were those that had been continuously propagated in culture prior to inoculation and ‘fresh’ isolates were taken from either human campylobacteriosis patients or chickens with minimal passage. Further details of this classification can be found in the original paper [30]. The ‘fresh’ isolates demonstrate the distinctive ‘all-or-nothing’ response described in §3.1, suggesting that considerably more transmission occurred for these isolates (figure 4).

**Figure 5.**
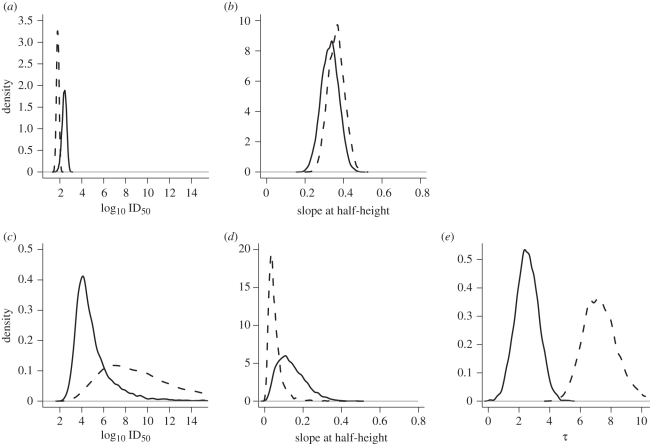
Effect of passage on posterior estimates of transmission and dose–response of *C. jejuni.* Comparison of the posterior estimates of dose–response and transmission for different samples of *C. jejuni* previously analysed by Chen *et al.* [30]. Model posterior distributions are plotted for laboratory-adapted ‘lab’ (solid black line) and ‘fresh’ isolates (dashed black line). Marginal posterior distributions for the ID_50_ (*a*,*c*) and slope at half-height on the log-scale (*b*,*d*) for the two groups based on the standard hyper-geometric dose–response model (*a*,*b*) and the combined dose–response and SI model (*c*,*d*), with posterior distribution of the total transmission *τ* (*e*). Without considering transmission, the ID_50_ for the ‘fresh’ isolates appears to be higher than for the ‘lab’ isolates. When transmission is taken into account this result is reversed, a consequence of the greater total transmission estimated for the ‘fresh’ isolates. However, as a consequence of the additional variability introduced by transmission, the posterior distributions for both the ID_50_ and the slope at half-height are overlapping and we cannot distinguish a difference in the dose–response for these two groups from these data. Posterior distributions are smoothed densities based on 20 000 MCMC samples and uniform priors.

Chen *et al.* used a hierarchical Bayesian approach to account for both the inter- and intra-isolate variation within the pooled data samples but treated co-housed animals as independent, neglecting the possible impact of transmission. Of the three datasets analysed in this paper, the uncertainties surrounding the experimental conditions of these data make them the least appropriate for our analysis. However, given the importance of this study as the primary published dose–response model for *Camyplobacter* spp. in chickens, it is important to consider how accounting for transmission may modify their conclusions [30]. The critical variable in terms of quantifying the potential for transmission is the group size. All of the dose groups reported in Chen *et al.* were housed in groups of 10 birds in negative pressure isolators, all of which received the same inoculating dose (S. A. Cawthraw 2010, personal communication). For simplicity and consistency with the rest of the paper, we replicate their analyses using a standard Bayesian methodology, which folds all of the variation in the data into the final posterior distribution. Our estimates using the hypergeometric dose–response model—assuming no transmission—demonstrate the same qualitative results as Chen *et al.* with a lower ID_50_ and smaller inter-host variability for the ‘fresh’ group (figure 5*a,b*). In order to avoid introducing bias into the estimate of transmission, we only include dose points where the final colonization status of all 10 birds in a group were reported (curated data tables are presented in the electronic supplementary material, technical appendix).

Visual inspection of the raw proportionate response suggests that more transmission has occurred in the groups challenged with ‘fresh’ isolates (figure 4), which is consistent with an estimated total transmission of more than two and half times that from the laboratory isolates (figure 5*e*, point estimates of *τ* = 7.22 ‘fresh’, compared with *τ* = 2.52 ‘lab’). With these estimated values of transmission, the qualitative comparison in the individual dose–response is reversed when compared with Chen *et al.*'s anaylsis, with a higher ID_50_ and larger inter-host variability for the ‘fresh’ group (figure 5*c,d*). Given the small number of replicate groups, we should take some caution in interpreting these data. In the presence of such an apparently large amount of transmission, it is unclear as to what extent the true geometrical shape of the individual dose–response (characterized by the ID_50_ and slope-at-half height) can be inferred. Indeed the overlapping posterior distributions for both the ID_50_ and slope at half-height under the HSI model suggest that we cannot distinguish the dose–response relationships for these two groups based on these data. Steep individual dose–responses and high rates of transmission will only be differentiated within our likelihood framework by the variation between replicate experiments. For the ‘fresh’ group only two of the included dose groups demonstrate a response that is not ‘all-or-nothing’. Furthermore, each of these groups also belongs to a different isolate of *C. jejuni*, each of which may be characterized by different dose–response relationships.

Chen *et al.* could estimate both the inter- and intra-isolate variability as they assumed that each individual bird constituted an independent sample from the dose–response distribution [30]. As the hosts were co-housed with the potential for transmission to occur then we should treat the data as coming from groups, dramatically reducing the number of replicate data points for each dose to one. The uncertainty this produces is immediately apparent from cursory inspection of the width of the posterior distributions for the combined model when compared with the individual model (figure 5). In the next section, we quantify this impact of transmission, in terms of the maximum *statistical information* we can obtain from a given experiment.

## 5. Impact of transmission on the information content of a dose–response experiment

In the previous section, we demonstrated that in principle we can use our combined likelihood to estimate dose response (*α*,*β*) and transmission (*τ*) parameters simultaneously using standard maximum likelihood or Bayesian MCMC approaches. The prospect for identifying the relative roles of transmission and the individual dose–response will be limited by the shape of the individual dose–response relationship, the size of the group and the number of hosts available for replicate experiments. However, transmission within groups has further implications for the statistical information that can be obtained from a given experiment beyond the systematic bias in the ID_50_ and slope at half-height. In this section, we will use some simple concepts from information theory to quantify how the number of hosts required to obtain a fixed quantity of information scales with transmission and group size.

We can quantify the information content of a single experimental observation, with *N* outcomes occurring with probability *p*_*i*_, in terms of the Shannon entropy:5.1
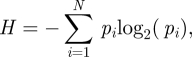
where log_2_ (*p*_i_) is the base 2 logarithm. Originally developed in the context of communication theory [56], entropy quantifies the intuitive notion that outcomes which are more uncertain can encode more information upon repeated observation. By increasing the likelihood of observing dose groups with maximal colonization, transmission bleeds information out of a given experimental system reducing the amount of information available for inference.

Let us consider the situation where we have a fixed number of animals *N*_tot_ available for an experiment. In the absence of transmission, the final colonization state of each bird constitutes a replicate observation with a binary outcome of *success* or *failure*. The maximum information for individual dose–response experiments (*H*_I_) will be obtained when the probability of colonization (success) is 1/2:5.2
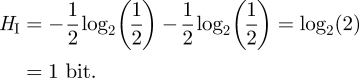


Each host will therefore contribute a maximum of 1 bit (log_2_ units) of information. Since entropy is additive by definition (equation (5.1)), an individual dose–response experiment with *N* animals will provide at most, *N* bits of information.

If we split the *N*_tot_ up and perform experiments in groups of *N* hosts—thus introducing the possibility of transmission between the co-housed hosts—we must treat each group as a replicate observation with *N*+1 possible outcomes. The maximum entropy for a group of size *N* is given when the probability of each outcome is uniform (*p*_*i*_ = 1/(*N*+1)):5.3
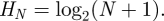


Given a fixed number of available hosts *N*_tot_, the maximum number of replicate groups will be *N*_tot_/*N*, giving a maximum information content for the group dose–response experiment of:5.4
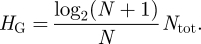


For group sizes greater than *N* = 1, *H*_G_ < *H*_I_ and therefore individual dose–response experiments will always be optimal in terms of maximizing the entropy (and therefore the total information) obtainable given a fixed number of hosts.

**Figure 6.**
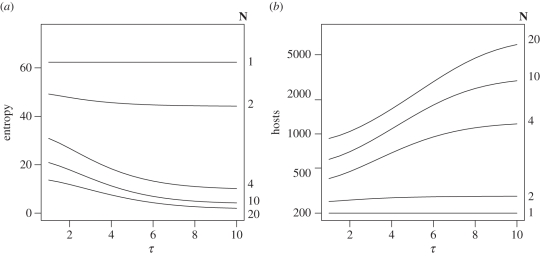
Quantitative impact of transmission on information content. Quantification of the impact of transmission for an experimental design with 10 dose groups *D*_*i*_ = 10^1^, 10^2^, 10^3^, 10^4^, 10^5^, 10^6^, 10^7^, 10^8^, 10^9^, 10^10^ and a fixed number of available hosts *N*_tot_ = 200. The individual dose–response is assumed to be hyper-geometric with *α* = 0.20, *β* = 125. Entropy is a measure of the global information content for a given experimental design (see main text for definition), plotted here as a function of the total transmission *τ* for five group sizes *N* = 1,2,4,10,20 (*a*). Since entropy is additive, we can calculate the number of additional hosts that would be have to be used to obtain the same information that would be obtained from an individual housing experiment (*b*). As the group size (*N*) and total amount of transmission (*τ*) increase, we need to use dramatically more animals in order to obtain the same results.

In practice, the maximum information obtainable from a given experimental system will also be limited by the form of *P*_inf_ and the choice of dose points *D*_*i*_. In figure 6*a*, we illustrate the relationship between transmission (*τ*) and entropy for our exemplar dose–response model (figure 1) under a fixed experimental design with 10 dose points *D*_*i*_, *N*_tot_ = 200 hosts and five group sizes *N* = 1,2,4,10,20.

It should first be noted that the entropy of a group dose–response experiment is less than for housing of birds individually even if transmission does not occur (*τ* = 0). This is determined by how we choose to interpret the response data: the possibility that transmission occurs—even if in practice it does not—reduces the amount of information that we can obtain from our data. As transmission increases, entropy decreases at a diminishing rate. In small groups, high values of *τ* will saturate the output leading to maximal colonization whenever a single member of a group becomes colonized after inoculation. This ‘all-or-nothing’ result will, therefore, constitute a lower bound on the global information that is obtainable under a given experimental design.

Although theoretically appealing, entropy is a rather abstract concept for communicating the efficiency of an experimental design. However, we can use the additivity property of entropy to quantify the relative efficiency of individual and group housing in more concrete terms. An individual dose–response experiment performed with *N*_tot_ birds provides *H*_I_ units of information. Given that a group housing experiment necessarily provides less information for the same number of birds (*H*_G_), we must perform more experiments. There will, therefore, be an excess number of hosts which must be killed in a group-housing experiment in order to obtain the same statistical information. The additivity property of entropy means that this excess number of hosts will simply be given by (*H*_G_/*H*_I_) *N*_tot_ (figure 6*b*). Once again as *τ* → ∞, there will be an upper bound on the excess number of hosts required which is increasing with the group size *N*. For a group size of 10, typical for experimental studies in *C. jejuni* in broilers, the number of excess birds required more than doubles even for the lower estimates of *τ* (≈1–2) estimated for 1 day-old chicks.

## 6. Discussion

Dose–response experiments are routinely used to characterize the relationship between an infectious agent and its host. In particular, the ID_50_ quantifies the relative infectiousness of different agents, and the slope of the response curve provides information on the heterogeneity between hosts. Graphical analyses, or the use of individual dose–response models, on dose–response data from co-housed hosts will lead to an estimate of the ID_50_ which is lower than the ‘true’ individual response. While the potential for this bias has been acknowledged in previous studies, in this paper, we quantify the nature and extent of this bias for the first time, to our knowledge, and use our new framework to reinterpret previously published dose–response data for *Campylobacter* in chickens. We have extended standard dose–response models to estimate individual dose–response and transmission parameters simultaneously using standard likelihood-based techniques. More generally, by interpreting the likelihood for our combined dose–response and transmission model using information theory, we can quantify the relative efficiency of different experimental designs.

The magnitude of the systematic bias of the ID_50_ owing to transmission depends on the shape of the individual dose–response, the group size, the rate of transmission and the length of the experiment. The shallower the individual dose–response is and the longer the experiment lasts, the greater the potential for bias owing to transmission to occur. However, this systematic bias is secondary to the commensurate increase in the variability of the outcome of individual replicate groups. Animals that are individually housed can each be considered as a single experimental replicate (with a binary response variable). When animals are housed in groups then the colonization status of each individual animal is theoretically correlated with the status of all other members of the group. Even when transmission does not in fact occur, the potential for transmission means that we must treat the group itself as the experimental replicate (with response variable taking *N*+1 values) instead of the individual.

Analysing group dose–response data with individual dose–response models will therefore greatly underestimate the uncertainty in estimates of the ID_50_. This is clearly demonstrated by our re-analysis of the data published by Chen *et al.* [30] presented in figure 5. Applying the individual hypergeometric model to these data leads to tight posterior distributions for the ID_50_ of the order of a single log_10_ (figure 5*a*), while the posterior distributions from the combined HSI model range over 12 log_10_ (figure 5*b*). This reflects a fundamental identifiability issue between the relative contributions of transmission and the individual dose–response. The statistical signature of transmission comes from the variability between replicates. Specifically, transmission increases the dispersion in the observed proportion of responding hosts at a given dose. In order to account for the transmission between co-housed hosts accurately requires the performance of multiple replicate experiments at the same dose-level. With only single replicate groups at a given dose, transmission generates ‘all-or-nothing’ empirical dose-curves that may be as equally well described by a shallow dose–response and high levels of transmission, as by a steep individual response with no transmission.

Group housing has been routine in dose response studies with avian species primarily owing to the recommendations of health and welfare regulations for social animals. However, such designs also have considerable practical and economic benefits, with the per-bird costs associated with individual housing of animals typically being comparable, or exceeding, the total costs associated with groups of co-housed birds. The primary goal of dose response studies is to estimate the ID_50_ for a given host–pathogen system to a given level of accuracy—typically within a single log_10_. As with any form of animal experimentation, it is desirable to achieve this with the minimum cost in animal lives. By this criteria, individual housing will always provide the optimal experimental design in terms of the number of host animals that must be killed to achieve the same experimental outcome. Our combined HSI model can correct the individual dose–response as observed in group experiments but can only achieve the same level of accuracy as an individual assay by greatly increasing the total number of animals that are killed (figure 6*b*).

Dose–response studies are also frequently used to compare the infectivity of different infectious agents [10,12,13,30] and the impact of different treatments or properties of the host [5,11]. The potential for transmission between co-housed hosts will greatly increase the number of repeat experiments necessary to see a significant difference between two groups, a factor that must be considered in power calculations when such experiments are being designed. The practical, economic and ethical advantages of group housing must be offset against the necessity to perform additional repeat experiments in order to achieve the same experimental accuracy. The ‘optimum’ experimental design will depend on the specific criterion for optimization (e.g. the total number of animals, number of groups) and the specific scientific questions, which are of interest.

However, there is a potential advantage to performing dose–response experiments in groups suggested by the HSI model presented in this paper. In terms of understanding the spread of infectious agents at a population level, the transmission process is arguably more important than factors affecting susceptibility. Population-level estimates of transmission cannot mechanistically distinguish between the relative contributions of susceptibility and transmissibility to the average rate of spread of an infectious agent. By performing dose–response studies in groups, we can simultaneously estimate the transmissibility of an infectious agent as well as the susceptibility of the host, separating out the mechanistic impact of a particular treatment. Should we wish to estimate both transmission rates and the dose–response relationship for a host–pathogen, a combined dose–response and transmission study may be more efficient than carrying out separately designed experiments. The design of such experiments will be critically dependent on the duration of the final end-point. There will be an inevitable tension between increasing the duration of the final sample point to improve estimates of transmission and reducing it to obtain a clearer estimate of the dose–response relationship. This relationship can be explored using the formal theory of experimental design [57], however doing so would require a more explicit accounting of the latent period between inoculation and the ability to detect colonization than is allowed by our current framework.

In this study, we have abstracted away the time-dependence between inoculation and detection through the concept of the total transmission (*τ*). It would be a natural extension of this work to derive models that explicitly incorporate a latent period. The experimental methodology of Ringoir *et al.* [11], combining dose–response studies with the intensive sampling of a transmission study, offers the potential to perform inference based on such models. Although we do not do so here, it would be possible, in principle, to perform inference using a model including a latent class using data-augmented MCMC [58]. However, it is also likely that the latent period is itself dose-dependent [23,25]—with larger doses reducing the time for a colonized host to begin shedding bacteria to the environment. Increasing the sophistication of the transmission model brings with it the necessity to estimate more parameters from what is typically sparse data. Given the identifiability issues that already exist for our current model, we believe that the support of more sophisticated models will require additional data from de novo experiments and is thus beyond the scope of the current study.

Implicit in our description of the transmission process as an SI model is a further assumption that there is no heterogeneity in the susceptibility of hosts to becoming colonized through transmission. At first glance, this assumption is at odds with the variability between hosts on inoculation that is explicitly modelled by the hypergeometric dose–response relationship. Given a heterogeneous population, we would *a priori* expect that hosts which fail to respond to a given inoculation are on average likely to be less susceptible compared with those that do. Thus, our estimates of transmission may be expected to be systematically lower than those demonstrated by seeder bird transmission studies. However, given that the ceacal contents of colonized birds typically contain orders of magnitude greater numbers of bacteria than the typical challenge dose, we believe that this is a reasonable assumption for these data where colonization is represented by a binary outcome. In reality, the infectious pressure of individual birds will slowly increase after inoculation with the load of bacteria. Heterogeneity in susceptibility could result in colonized hosts effectively becoming infectious to different co-housed hosts at different times dependent on their individual level of susceptibility. Accounting for such heterogeneity between hosts is likely to be essential for models incorporating latency in order to properly distinguish between latency and early infection events.

In this paper, we re-visit two studies, which compare properties of the host [11] and the infectious agent [30] for *C. jejuni* in chickens. Given the small number of replicates performed in these experiments, we must be cautious in interpreting the results. However, our analysis suggests that the differences between treatment groups in these two studies can be equally well explained by changes in transmissibility of *C. jejuni* when compared with susceptibility of the host as was previously reported.

Analysis of the pooled data of Chen *et al.* [30] suggests that total transmission is greater in experiments with ‘fresh’ isolates when compared with those which had been repeatedly passaged in laboratory conditions. However, the greater ‘total transmission’ (*τ*) for the ‘fresh’ group of isolates is not necessarily evidence of higher transmission rates. In the interests of parsimony, we model transmission using an SI epidemic model. This is an approximation, as it assumes that individuals who are successfully colonized after inoculation will immediately become infectious. In reality, there is likely to be a latent period between inoculation and infectiousness dependent on the internal dynamics of the infectious agent [15]. The greater value of *τ* for the ‘fresh’ isolates may equally be well explained by a faster rate of replication within the host leading to a shorter latent period and therefore a shorter time between inoculation and the first host becoming infectious. Since we have no information on the time course of infection from standard dose–response experiments, it is impossible to distinguish between these two hypotheses.

Our analysis of the data of Ringoir *et al.* [11] suggests that transmission rates of *C. jejuni* are higher in 14 day-old birds when compared with day old chicks, despite the minimum effective infectious dose ID_50_ being over a log_10_ higher. A putative increase in susceptibility of birds to *C. jejuni* with age has been discussed by previous authors as a possible mechanism for the *C. jejuni* negative ‘lag phase’ of 14–21 days reported in commercial flocks [42]. However, evidence for this hypothesis within the literature is lacking. Indeed, Ringoir *et al.*'s data suggest that younger birds are in fact more susceptible to colonization than older birds, rather than less. A similar challenge study carried out by a different research group demonstrated no clear difference in susceptibility between chicks and older birds, but a pronounced increase in the rate of onset of bacterial shedding post-inoculation [59]. Within our model framework, an increase in the rate of onset will manifest itself as a increase in the estimated net rate of transmission. Thus, taken together with our analyses, we believe that these data are more consistent with the alternative hypothesis that it is changes in transmissibility rather than susceptibility that underlie the ‘lag phase’. Sampling of commercial flocks can only detect colonization above a certain threshold determined by the number of birds sampled. Infrequent exposure of chicks to environmental sources of *Campylobacter* could lead to colonization in a small number of chicks below this threshold which do not excrete sufficient quantities of contaminated faecal matter to efficiently transmit to other members of the flock. *Campylobacter jejuni* would therefore only become detectable when the birds aged sufficiently, and the rate of transmission increases sufficiently that invasion of the flock could take place.

This paper has focused on *C. jejuni* in chickens owing to the availability of data and simple (SI) transmission dynamics for this system, where the total transmission over an experiment can be quantified in terms of a single parameter (*τ*). However, our key observation—that the over-dispersion of response data can be used to infer rates of transmission within groups—is likely to be applicable to more general host–pathogen systems with more complex transmission dynamics, with the attendant requirements for more detailed and extensive data. Furthermore, using information theory, we have demonstrated the general result that individual housing of host animals is an optimal experimental design for the quantification of dose–response relationships. When individual housing is not practically or economical possible, then multiple replicates of small groups are preferable to single replicates of larger groups.
